# Effect of Fluorosis on Liver Cells of VC Deficient and Wild Type Mice

**DOI:** 10.1155/2014/287464

**Published:** 2014-02-16

**Authors:** Wei Wei, Yan Jiao, Yonghui Ma, John M. Stuart, Xiudian Li, Fusheng Zhao, Lishi Wang, DianJun Sun, Weikuan Gu

**Affiliations:** ^1^Center for Endemic Disease Control, Centers for Disease Control and Prevention, Institute of Endemic Fluorosis Disease, Harbin Medical University, Harbin, Heilongjiang 150081, China; ^2^Departments of Orthopaedic Surgery and BME, Campbell Clinic, and Pathology, University of Tennessee Health Science Center (UTHSC), 956 Court Avenue, Memphis, TN 38163, USA; ^3^Mudanjiang Medical College, Mudanjiang, Heilongjiang 157001, China; ^4^Division of Connective Tissues Diseases, Department of Medicine, University of Tennessee Health Science Center, Memphis, TN 38163, USA; ^5^Department of Veterans Affairs Medical Center, Memphis, TN 38104, USA; ^6^Department of Nephrology, Second Affiliated Hospital of Heilongjiang University of Chinese Medicine, Harbin, Heilongjiang 150001, China

## Abstract

For decades, mouse and other rodents have been used for the study of oxidative or related studies such as the effect of fluoride. It is known that rodents normally synthesize their own vitamin C (VC) due to the presence of a key enzyme in ascorbic acid synthesis, l-gulono-lactone-**γ**-oxidase (*Gulo*), while humans do not have the capacity of VC synthesis due to the deletion of most parts of the GULO gene. The spontaneous fracture (*sfx*) mouse recently emerged as a model for study of VC deficiency. We investigated the effect of fluoride on liver cells from wild type Balb/c and *sfx* mice. We found that activities of SOD, GPx, and CAT were reduced in both wild type and *sfx* mice; however, the amount of reduction in the *sfx* cells is more than that in Balb/c cells. In addition, while both cells increased MDA, the increase in the *sfx* cells is greater than that in Balb/c cells. Gene networks of *Sod*, *Gpx*, and *Cat* in the liver of humans and mice are also different. Our study suggests that reaction to fluoride in vitamin C deficient mice might be different from that of wild type mice.

## 1. Introduction

Human and mouse genes are 99% the same; however, a few important differences, including the vitamin synthesis, should have been paid much attention, particularly for the biomedical research. Our study intends to explore the answer of whether the wild type mouse model is the right choice for the study of fluoride and, to certain extent, the oxidative stress. To date, much evidence strongly indicates that fluorosis is closely related to oxidative stress [1–5]. Furthermore, study of oxidative stress using mouse model has been widely accepted. An important issue related to mouse model is that vitamin C (VC) was suggested to reduce oxidative stress as one of the antioxidants [4, 6, 7]. It is known that mice normally synthesize their own vitamin C due to the presence of a key enzyme in ascorbic acid synthesis, l-gulono-lactone-*γ*-oxidase (*Gulo*), which is present in the liver. However, this enzyme is lacking in humans. Because mice can synthesize VC, it is not considered an essential dietary component for this species, and therefore VC is not usually added to mouse chow. In contrast, humans depend entirely on dietary supplementation for their VC.

For decades, the utilization of the mouse or other rodents for study of oxidative or related studies such as fluoride as the animal model has never been challenged. As of August 5, 2013, using the two key words “fluoride” and “mouse,” we found 1674 publications from PubMed. When we restricted the search for the year of 2013 only, we found 46 publications. Using key words “oxidative mouse,” we found 26606 publications from PubMed. Among these publications 2008 are from 2013, but none of them apparently are using the VC deficiency mice. Our recent study indicates that there are different gene expression levels between wild type mice and VC deficient *sfx/sfx* mice, which were supplied with sufficient quantities of vitamin C [8, 9]. One key question is whether the differential expressions between wild type and VC deficient mice lead them to react differently to environmental stimulations such as by oxidative toxic components.

To explore the difference *in vitro* between cells from *sfx* and wild type mice, we investigated the effect of fluoride on liver cells from wild type and *sfx* mice. We assume that using liver cells from *sfx/sfx* mouse of fluorosis may allow us to have a better understanding of the mechanism of fluorosis without the disturbance of vitamin C. Thus, we intended to provide the direct evidence that wild type mice are not a good model to study human oxidative pathways. Particularly, we examined the differences in oxidative stress indexes between cells from wild type Balb/c and *sfx/sfx* mice.

## 2. Materials and Methods

### 2.1. Cells Culture

Hepatocytes were isolated from Balb/c and *sfx/sfx* mice with a body weight range of 20–35 g using the two-step hepatic portal vein perfusion technique [10]. Hepatocytes were cultured on rat tail collagen type I at a density of 5 × 10^5^ cells/mL. Primary hepatocytes were maintained in a HepatoZYME-SFM medium (Invitrogen, USA), supplemented with 200 U/mL penicillin, 100 *μ*g/mL streptomycin (Sigma-Aldrich, USA), and 10% fetal bovine serum (Mediatech Inc., VA, USA) at 37°C in a humidified atmosphere of 95% air/5% CO_2_. The medium was changed after optimal attachment. Medium contains RPMI-1640 medium (HyClone, Utah, USA), supplemented with 200 U/mL penicillin, 100 *μ*g/mL streptomycin, and 2% fetal bovine serum. In addition, the medium was introduced in 12.5 *μ*M of vitamin C and contained NaF at final concentrations of 0, 0.25, 0.5, 1, 2, 4, and 8 mM, respectively. The cells were incubated for 1, 2, 4, 6, 12, 24, 48, and 72 h. All experiments were performed in triplicate.

### 2.2. Cell Growth and Viability Assay

Hepatocytes were cultured in 96-well plates at a density of 2 × 10^4^ cells/well. After allowing the cells to adhere, they were treated for 1, 2, 4, 6, 12, 24, 48, and 72 h with the indicated concentrations of vitamin C and NaF. WST-8 (Cell Counting Kit-8/CCK-8, Sigma-Aldrich, MO, USA) assays were performed to quantify LS8 cell viability after exposure to the different concentrations of vitamin C and NaF, respectively. The absorbance was measured at 450 nm using an Absorbance Microplate Reader (SpectraMax 340PC384, USA). Five samples were analyzed and the mean value was calculated for each NaF concentration and time point.

### 2.3. Protein Preparation and Determination

Hepatocytes were cultured in 25 cm^2^ flasks at a density of 2 × 10^6^ cells/flask. After NaF treatment, the hepatocytes were harvested by the rubber policeman, then resuspended in 50 mM Tris buffer containing 0.5% Triton X-100, pH 8.0, and lysed by freeze thawing. Lysates were centrifuged at 6,000 ×g for 15 min at 4°C. The supernatant obtained was used for the SOD, CAT, and GPx activities measurements. The supernatant was then removed and stored on ice. The protein content of the supernatant was determined using Thermo Scientific NanoDrop 2000 spectrophotometers (Thermo Fisher Scientific NanoDrop Products, Wilmington, Delaware, USA).

### 2.4. SOD Assay

Superoxide dismutase (SOD) activity was measured using the SOD determination kit-WST (Sigma-Aldrich, Buchs, Switzerland). As an inhibition activity, the SOD activity was quantified by measuring the developed color at 450 nm using the Absorbance Microplate Reader (SpectraMax 340PC384, USA). All operations are following the manufacturer's instructions. The SOD activity was calculated as an inhibition rate percentage and expressed as units (SOD activity (units) = SOD activity (inhibition rate)/(1-SOD activity (inhibition rate)).

### 2.5. CAT Assay

Hepatocytes catalase (CAT) activity was determined using the OxiSelect Catalase Activity Assay Kit (colorimetric method) (Cell Biolabs, San Diego, CA, USA). It records the decomposition of H_2_O_2_ by decreasing the absorbance at 520 nm. One unit of CAT activity is equal to the amount of enzyme(s) that decomposed 1.0 *μ*mole of H_2_O_2_ per minute at 25°C. The CAT activity was calculated and expressed as U per mg protein.

### 2.6. GPx Assay

Glutathione peroxidase (GPx) activity was measured with the Glutathione Peroxidase assay kit (Cayman Chemical, Ann Arbor, MI, USA) in accordance with the manufacturer's instructions. Measurement of GPx activity was performed according to the colorimetric Method that is based on the monitoring of the oxidation of NADPH per minute at 25°C. The reaction rate can be determined using the NADPH extinction coefficient of 0.00373 *μ*M^−1^ after adjusting for the path length of the solution in the well (0.6 cm). The absorbance was measured at 340 nm using the Absorbance Microplate Reader (SpectraMax 340PC384, USA) and the concentration was expressed in nmoles GPx/min/mg protein.

### 2.7. Measurement of MDA

Hepatocytes were suspended at 1 × 10^7^ cells/mL in PBS containing butylated hydroxytoluene for malondialdehyde (MDA). The hepatocytes were sonicated in phosphate-buffered saline containing butylated hydroxytoluene for MDA. The MDA in the whole homogenate was measured using the OxiSelect TBARS assay kit containing thiobarbituric-acid-reactive substances (Cell Biolabs, San Diego, CA, USA). All standards and samples were assayed in duplicate in accordance with the manufacturer's instructions. The developed color was read at 532 nm using the Absorbance Microplate Reader (SpectraMax 340PC384, USA). TBARS formation was expressed as *μ*M TBARS 10^6^ cells^−1^.

### 2.8. Association of *Gulo* with Oxidative Genes

In order to understand the relationship between *Gulo* and genes of Glutathione peroxidase (*Gpx*), superoxide dismutase (*Sod*) and hepatocytes catalase (*Cat*) in liver, we examined the gene network among *Gulo* and three oxidative genes. Because gene network is built upon the expression of genes in multiple strains, we were not able to use the *sfx* mice (a single strain) with that of Balb/c for gene network construction. Therefore, we took the advantage of available gene expression data of multiple mouse strains and a human population in GeneNetwork (http://www.genenetwork.org/webqtl/main.py). We examined the association of gene expression between *Gulo* and three oxidative stress genes in this study using gene expression profiles of mouse recombinant inbred (RI) strains generated using Affymetrix Array M430A by investigator at UCLA [11] and of the Human Liver Cohort [12, 13] which includes 427 human liver samples on a comprehensive gene expression microarray. The data has been deposited at GeneNetwork.

### 2.9. Statistical Analysis

All results were expressed as mean ± SEM. The data were analyzed for statistical significance using Student's *t*-test. *P* values less than 0.05 were considered statistically significant. All results were presented with histograms in Microsoft Excel.

## 3. Results and Discussion

### 3.1. The Viabilities of Hepatocytes from Balb/c and *sfx/sfx* Mice with Vitamin C

Cell viability was evaluated by the WST-8 assay. We first examined the viabilities of hepatocytes after treatment with vitamin C. We intended to identify the concentrations of vitamin C, which led to the same cell viability of hepatocytes from *sfx/sfx* and Balb/c mice. The results suggest that the cell viability of hepatocytes in the two types of mice is consistent at 12.5 *μ*m vitamin C from 1 h–72 h, at each time point (data not shown). Accordingly, we used 12.5 *μ*m vitamin C in the late experiments.

### 3.2. The Viabilities of Hepatocytes from Balb/c and *sfx/sfx* Mice after Treatment with NaF

**Figure 1:**
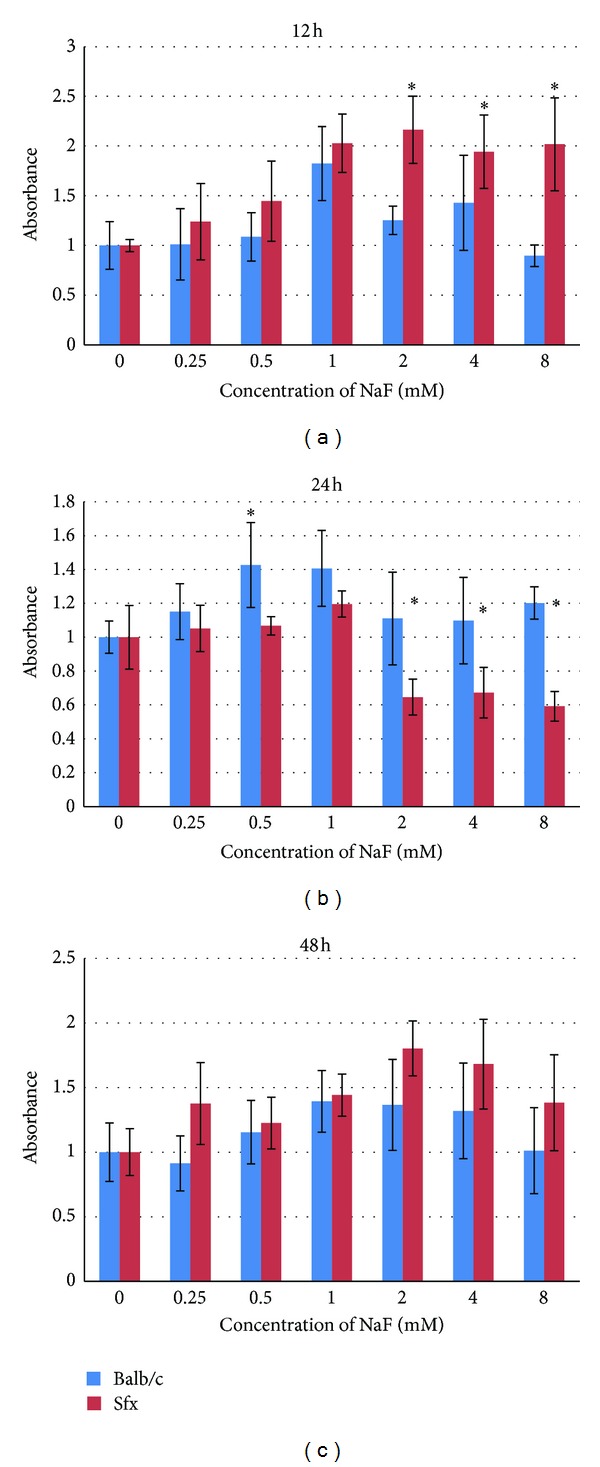
The viabilities of hepatocytes at three time points from Balb/c and *sfx/sfx* mice after treatment with NaF. *x*-axis: concentration of NaF (mM); 3′ UTR. *y*-axis: absorbance.

Cell viability test results of the hepatocytes from Balb/c and *sfx* mice for NaF concentrations after 12, 24, and 48 hrs were shown in histogram in Figure 1. In this experiment, the control groups of two types are calculated, respectively, as reference in the cell viabilities of all experimental groups. In 48 hours, because the viabilities of the control group of two types decreased with different degrees compared to 24 hours, the percentage of cell viabilities increased in some experimental groups. In this case, cell viability data showed improvement in 48 hours.

The results show that the cell viability in two types of cells had a significant difference with higher concentration at 12 and 24 hrs. However, the cell viability between *sfx* and Balb/c between these two time points showed opposite results. At 12 hrs, the cell viability of 2, 4, and 8 mM of *sfx* mice is much better than that of Balb/c, with *P* values of 0.0005, 0.09, and 0.0008, respectively. In contrast, at 24 hrs, the cell viability of 2, 4, and 8 mM of *sfx* mice is much less than that of Balb/c, with *P* values of 0.007, 0.01, and 5.77846*E*−06, respectively. This data seemingly suggests that there is a difference in the time-sequence of the response to NaF between cells from *sfx* and wild type.

In addition, at 48 h, the cell viability did not show a significant difference between those two types of cells, with *P* values of 0.04, 0.15, and 0.13 at concentrations of 2, 4, and 8 mM, respectively. Again this may be the net result of opposite reactions at 12 and 24 hrs between cells from *sfx* and wild types.

### 3.3. Effects of NaF on Antioxidant Status

**Figure 2:**
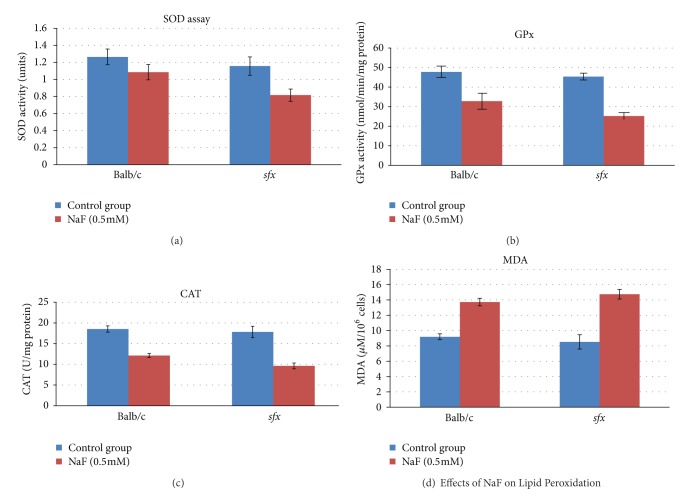
Effects of NaF on antioxidant status. *x*-axis: cells from different mice in treatment. *y*-axis: units of enzyme activity. (a) SOD activity was measured using the SOD determination kit. The SOD activity was calculated as an inhibition rate percentage and expressed as units (SOD activity (units) = SOD activity (inhibition rate))/(1-SOD activity (inhibition rate)). (b) CAT activity was determined using the OxiSelect Catalase Activity assay kit. One unit of CAT activity is equal to the amount of enzyme(s) that decomposed 1.0 *μ*mole of H_2_O_2_ per minute at 25°C. The CAT activity was calculated and expressed as *U* per mg protein. (c) GPx activity was measured with the Glutathione Peroxidase assay kit. The absorbance was measured at 340 nm using the Absorbance Microplate Reader (SpectraMax 340PC384, USA) and the concentration was expressed in nmoles GPx/min/mg protein. (d) Effects of NaF on lipid peroxidation. The MDA in the whole homogenate was measured using the OxiSelect TBARS assay kit. The developed color was read at 532 nm using the Absorbance Microplate Reader. TBARS formation was expressed as *μ*M TBARS 10^6^ cells^−1^.

We measured the activities of superoxide dismutase (SOD), glutathione Peroxidase (GPx), and catalase (CAT) of hepatocytes from cells of Balb/c and *sfx* mice treated with 0.5 mM NaF for 48 hours. The results show that the SOD, GPx, and CAT activity are different between the control group and the NaF group in each of the two types of hepatocyte. Moreover, the differences between NaF treated and the control in *sfx* and wild type cells are also different, respectively (Figure 2). The main biological roles of SOD, GPX, and CAT are to protect the organism from oxidative damage. SOD is an enzyme that catalyzes the dismutation of superoxide into oxygen and hydrogen peroxide. Thus, they are an important antioxidant defense in nearly all cells exposed to oxygen. The biochemical function of GPx is to reduce lipid hydroperoxides and to reduce free hydrogen peroxide in water. CAT catalyzes the decomposition of hydrogen peroxide in water and oxygen.

In Balb/c cells, the activity of SOD in NaF (0.5 mM) treated hepatocytes is lower than that in control cells (Figure 2(a)). The *P* value is 0.022. In *sfx* cells, the activity of SOD in NaF (0.5 mM) treated cells is significantly lower than that in control cells, with *P* value of 0.0002. The difference in the net reduction of SOD activity between cells from Balb/c and *sfx* is 0.022. Thus, reduction of SOD activity in the *sfx* cells is much greater than that in Balb/c cells.

NaF (0.5 mM) treatment showed the greatest effect on the Gpx activity in hepatocytes (Figure 2(b)). In Balb/c cells, the activity of GPx in NaF (0.5 mM) treated cells is much lower than that in control cells. The *P* value is 8.10652*E*−06. In *sfx* cells, the activity of GPx in NaF (0.5 mM) treated cells is also significantly lower than that in control cells, with *P* value of 7.66305*E*−06. The difference in GPx activity between hepatocytes from Balb/c and *sfx* reached a significant level, with *P* value of 0.0016. Thus, amount of reduction of GPx activity in the *sfx* cells is much greater than that in Balb/c cells.

Changes of CAT activity are similar to that of SOD (Figure 2(c)). In Balb/c hepatocytes, the activity of CAT in NaF (0.5 mM) treated cells is much lower than that in control cells. The *P* value is 1.51688*E*−05. In *sfx* cells, the activity of CAT in NaF (0.5 mM) treated cells is also significantly lower than that in control cells, with *P* value of 2.31606*E*−08. The difference in CAT activity between cells from Balb/c and *sfx* also shows a significant level, with *P* value of 0.0297.

These data raise the question whether the study of the fluoride and oxidative stress or vitamin C relevant genes should use the *Gulo* deficient mouse model. Since the wild type mouse has the endogenous vitamin C synthesizing gene, which humans have lost during evolution. The result from wild type mice may not be as applicable to humans as that from *sfx* mice.

### 3.4. Effects of NaF on Lipid Peroxidation

Lipid peroxidation product MDA was measured in the NaF treated and wild type Balb/c hepatocytes. As shown in Figure 2(d), MDA content was significantly higher in NaF treated hepatocytes compared to control groups from both Balb/c and wild type controls, with *P* values of 8.56273*E*−06 and 1.25082*E*−07, respectively. Moreover, the amount of increase of MDA in NaF treated *sfx* hepatocytes is greater than that in Balb/c cells, with *P* value of 0.0095. Thus, the amount of net changes of MDA in hepatocytes shows opposite result in comparison with that of SOD, GPx, and CAT.

MDA is the end product of lipid peroxidation considered as a sensitive index to assess lipid peroxidation. The increased MDA and decreased SOD, GPx, and CAT in *sfx* mice agree with each other. The hepatocytes MDA levels of NaF groups were significantly increased, indicating the presence of enhanced lipid peroxidation due to NaF injury. Moreover, there are significant differences between cells from Balb/c and *sfx* mice in MDA quantity changes (in NaF group), indicating that there are differences between the two models in reactions to fluorosis.

### 3.5. Association of *Gulo* with GPx, *Cat*, and SOD Genes in Wild Type Mice in Human Population

From GeneNetwork, we identified 18 probes for mouse genes, *Gpx1*, *Gpx3*, *Gpx4*, *Gpx5*, *Gpx1*, *Gpx7*, *Gpx8*, *Gulo*, *Cat*, *Sod1*, *Sod2*, and *Sod3*. From data of human liver cohort, we identified 15 probes for genes GPX1, GPX3, GPX4, GPX5, GPX1, GPX7, GPX8, CAT, SOD1, SOD2, and SOD3. Because humans do not have *Gulo* gene, we first compared Spearmen rank correlation with/without *Gulo* in mice and humans separately. A third analysis was done with mouse genes including *Gulo*.

The correlations of expression levels among major oxidative genes are opposite between mice and humans (Supplementary Tables s1 and s2 in Supplementary Material available online at http://dx.doi.org/10.1155/2014/287464). For example, (1) in mice, the expression level of catalase is positively correlated to the expression level of *Sod2*, with *R* value of 0.678. In humans, the expression of catalase is positively correlated to that of GPX4 and SOD1, with *R* values of 0.5 and 0.8, respectively. Furthermore, catalase is strongly negatively correlated to SOD2, with *R* value of −0.7; (2) in mice, *Sod1* did not show strong correlation to other genes, while *Sod2* showed positive correlation with Gpx1 and Gpx4 with *R* values of 0.5 for both. In humans, SOD1 is negatively correlated to GPX1, GPX3, and SOD2, with *R* values of −0.51, −0.51, and −0.57.

**Figure 3:**
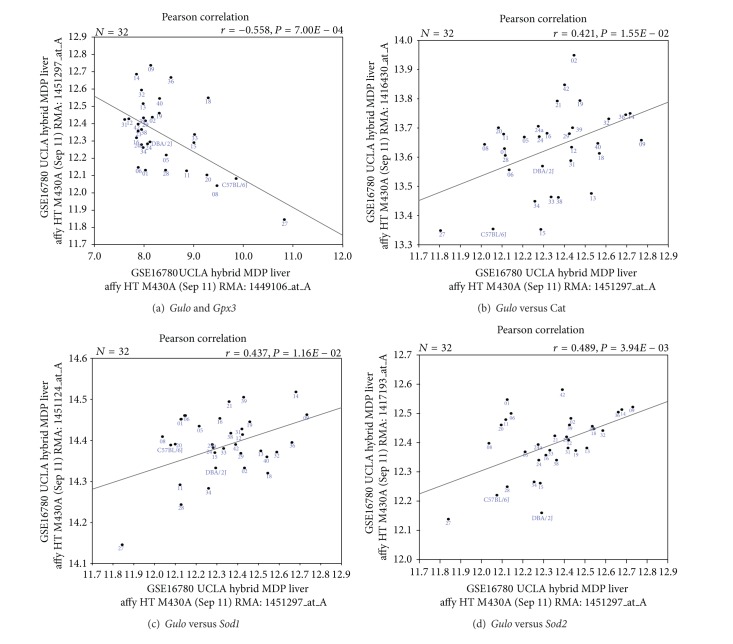
Correlation between expression level of *Gulo* and *Gpx3*, *Cat*, *Sod1*, and *Sod2* in mouse liver. Gene expression data are from GSE16780 UCLA Hybrid MDP Liver Affy HT M430A (Sep11) RMA database. (a) *x*-axis: Gpx3: glutathione peroxidase 3; 3′ UTR. *y*-axis: *Gulo*: gulonolactone (*L*-) oxidase (ascorbic acid biosynthesis; mid 3′ UTR). (b) *x*-axis: *Gulo* mid 3′ UTR. *y*-axis: *Cat* mid 3′ UTR 3. (c) *x*-axis: *Gulo* mid 3′ UTR. *y* -axis: *Sod1* first four exons and 3′ UTR. (d) *x*-axis: *Gulo* mid 3′ UTR. *y* -axis: *Sod2* last three exons and proximal 3′ UTR.

We next examined whether expression of *Gulo* is correlated to the expression of any of those genes in mice. We found that the expression level of *Gulo* is negatively correlated to one gene, the Gpx3, with *R* value of −0.558 (Figure 3). Furthermore, the expression of *Gulo* has a weak positive correlation with that of catalase, *Sod1*, and *Sod2* (Figure 3).

**Figure 4:**
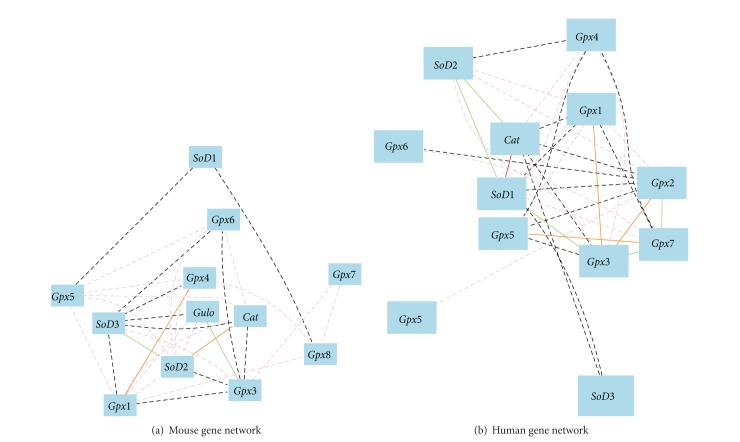
Gene networks of mice and humans. The 13 nodes in the graph show the selected traits. All nodes are displayed. The 47 edges between the nodes, filtered from the 78 total edges and drawn as curves, show Pearson correlation coefficients greater than 0.2 or less than −0.2. The graph's canvas is 40.0 by 40.0 cm, and the node labels are drawn with a 10.0 point font, and the edge labels are drawn with a 10.0 point font. (a) gene network of *Gulo* and oxidative genes in mice. (b) gene network of oxidative genes in human liver.

We finally constructed the gene network for both mice and humans. As shown in Figure 4, the gene networks between mice and humans are significantly different. In mice, four pairs of positive and negative associated genes form the core of the network. They are *Gulo* and *Gpx3*, *Sod3* and *Sod2*, *Gpx4* and *Gpx1*, and *Cat* and *Sod2*. In humans, the network core is composed of CAT and SOD1 with several GPX genes. Thus, the expression of *Gulo* at least partially influences the expression of those oxidative genes in mice which potentially lead to the difference in the pathways between non-*Gulo* humans and mice with *Gulo* gene.

The gene networks in this study are composed by using mouse RI strains and human population. Because the *Gulo* deficient mouse is a single strain, its gene network has not been constructed. Future study on the impact of *Gulo* deficient on the oxidative network *in vivo* will enhance our understanding of detailed molecular mechanism of oxidative pathway in *Gulo* deficient mice.

## 4. Conclusions

Our data show that there are significant differences in SOD, GPx, and CAT activity and MDA production when treated with fluoride between liver cells of Balb/c and the *Gulo* deficient *sfx* mice, indicating that there are differences between wild type and vitamin C deficient mice for the study of fluorosis. Comparison of gene coexpressions of these oxidative genes between mice and humans suggests that their molecular pathways are different. Taken together, we hypothesized that the wild type mice have inevitable shortcomings as a model to study human oxidative relevant disorders, as it has ignored the role of vitamin C in the oxidative pathway.

## Supplementary Material

Supplementary Table S1: Spearman rank correlation among mouse three oxidative genes. Mouse probes for three oxidative genes, Gpx1-Gpx8, Cat, and Sod1-Sod3 are all identified from BXD strains at geneNetwork. Spearman rank correlations are analyzed using GeneNetwork. Positive and negative correlations that are equal or higher than 0.5 and equal or lower than -0.5 are colored.
Supplementary Table S2: Spearman rank correlation of three oxidative genes among humans. Probes for human genes CAT, GPX1-GPX7, SOD1-SOD3 are all identified from data of whole genome gene expression of human liver at GeneNetwork. . Spearman rank correlations are analyzed using GeneNetwork. Positive and negative correlations that are equal or higher than 0.5 and equal or lower than -0.5 are colored.
