# Supplementary material for: Effect of Fluorosis on Liver Cells of VC Deficient and Wild Type Mice
Source: ScientificWorldJournal. 2014 Feb 16;2014:287464. doi: 10.1155/2014/287464 (PMC3947854; doi:10.1155/2014/287464)
Supplement: Supplementary file 1 — Supplementary Table S1: Spearman rank correlation among mouse three oxidative genes. Mouse probes for three oxidative genes, Gpx1-Gpx8, Cat, and Sod1-Sod3 are all identified from BXD strains at geneNetwork. Spearman rank correlations are analyzed using GeneNetwork. Positive and negative correlations that are equal or higher than 0.5 and equal or lower than -0.5 are colored. Supplementary Table S2: Spearman rank correlation of three oxidative genes among humans. Probes for human genes CAT, GPX1-GPX7, SOD1-SOD3 are all identified from data of whole genome gene expression of human liver at GeneNetwork. . Spearman rank correlations are analyzed using GeneNetwork. Positive and negative correlations that are equal or higher than 0.5 and equal or lower than -0.5 are colored. [file 287464.f1.pdf]

Supplementary Table s1. Spearman rank correlation among mouse three oxidative genes (Mouse without Gulo)

Spearman Rank Correlation (rho)

Pearson r

| | Trait1 | Trait2 | Trait3 | Trait4 | Trait5 | Trait6 | Trait7 | Trait8 | Trait9 | Trait10 | Trait11 | Trait12 | Trait13 | Trait14 | Trait15 | Trait16 | Trait17 |
|---|---|---|---|---|---|---|---|---|---|---|---|---|---|---|---|---|---|
| Trait 1: GSE16780_UCLA_ML0911::1416429_a_at_A<br>Cat on Chr 2 @ 103.294184 Mb<br>catalase<br>distal 3' UTR | ***n***<br>32 | 0.752<br>32 | 0.141<br>32 | -0.324<br>32 | 0.093<br>32 | 0.209<br>32 | 0.189<br>32 | 0.404<br>32 | -0.044<br>32 | -0.072<br>32 | 0.451<br>32 | -0.144<br>32 | 0.514<br>32 | 0.366<br>32 | 0.178<br>32 | -0.318<br>32 | -0.367<br>32 |
| Trait 2: GSE16780_UCLA_ML0911::1416430_at_A<br>Cat on Chr 2 @ 103.29421 Mb<br>catalase<br>mid 3' UTR | 0.754<br>32 | ***n***<br>32 | 0.237<br>32 | -0.289<br>32 | -0.040<br>32 | 0.035<br>32 | 0.231<br>32 | 0.180<br>32 | 0.107<br>32 | -0.010<br>32 | 0.349<br>32 | -0.044<br>32 | 0.589<br>32 | 0.464<br>32 | 0.237<br>32 | -0.023<br>32 | -0.172<br>32 |
| Trait 3: GSE16780_UCLA_ML0911::1460671_at_A<br>Gpx1 on Chr 9 @ 108.241871 Mb | 0.152<br>32 | 0.247<br>32 | ***n***<br>32 | -0.144<br>32 | 0.475<br>32 | 0.537<br>32 | 0.253<br>32 | -0.120<br>32 | 0.137<br>32 | 0.179<br>32 | 0.366<br>32 | -0.135<br>32 | 0.410<br>32 | 0.416<br>32 | -0.162<br>32 | 0.043<br>32 | -0.359<br>32 |

| | | | | | | | | | | | | | | | | | |
|---|---|---|---|---|---|---|---|---|---|---|---|---|---|---|---|---|---|
| glutathione peroxidase 1<br>exons 1 and 2 and proximal 3' UTR | | | | | | | | | | | | | | | | | |
| Trait 4: GSE16780_UCLA_ML0911::1449106_at_A<br>Gpx3 on Chr 11 @ 54.723291 Mb<br>glutathione peroxidase 3<br>3' UTR | -0.445<br>32 | -0.478<br>32 | -0.335<br>32 | ***n***<br>32 | -0.073<br>32 | -0.096<br>32 | -0.085<br>32 | -0.268<br>32 | 0.254<br>32 | -0.006<br>32 | -0.471<br>32 | 0.177<br>32 | -0.238<br>32 | -0.173<br>32 | -0.343<br>32 | 0.079<br>32 | 0.238<br>32 |
| Trait 5: GSE16780_UCLA_ML0911::1451695_a_at_A<br>Gpx4 on Chr 10 @ 45.595286 Mb<br>glutathione peroxidase 4<br>last five exons | 0.016<br>32 | 0.004<br>32 | 0.654<br>32 | -0.125<br>32 | ***n***<br>32 | 0.666<br>32 | 0.220<br>32 | 0.248<br>32 | -0.102<br>32 | 0.512<br>32 | 0.387<br>32 | -0.289<br>32 | 0.194<br>32 | 0.258<br>32 | 0.076<br>32 | 0.084<br>32 | -0.403<br>32 |
| Trait 6: GSE16780_UCLA_ML0911::1456193_x_at_A<br>Gpx4 on Chr 10 @ 45.595578 Mb<br>glutathione peroxidase 4<br>last exon and 3' UTR | 0.170<br>32 | 0.122<br>32 | 0.704<br>32 | -0.195<br>32 | 0.804<br>32 | ***n***<br>32 | 0.429<br>32 | 0.115<br>32 | -0.018<br>32 | 0.376<br>32 | 0.441<br>32 | -0.262<br>32 | 0.328<br>32 | 0.458<br>32 | -0.242<br>32 | -0.125<br>32 | -0.151<br>32 |
| Trait 7: GSE16780_UCLA_ML0911::1420698_at_A<br>Gpx5 on Chr 13 @ 21.378334 Mb<br>glutathione peroxidase 5<br>distal 3' UTR | 0.137<br>32 | 0.248<br>32 | 0.326<br>32 | -0.017<br>32 | 0.301<br>32 | 0.423<br>32 | ***n***<br>32 | 0.089<br>32 | -0.061<br>32 | 0.009<br>32 | 0.113<br>32 | -0.219<br>32 | 0.261<br>32 | 0.398<br>32 | -0.361<br>32 | 0.173<br>32 | 0.212<br>32 |

| | | | | | | | | | | | | | | | | | |
|---|---|---|---|---|---|---|---|---|---|---|---|---|---|---|---|---|---|
| Trait 8: GSE16780_UCLA_ML0911::1452135_at_A<br>Gpx6 on Chr 13 @ 21.410881 Mb<br>glutathione peroxidase 6 | 0.417<br>32 | 0.329<br>32 | -0.017<br>32 | -0.255<br>32 | 0.148<br>32 | 0.043<br>32 | 0.245<br>32 | ***n***<br>32 | -0.264<br>32 | 0.188<br>32 | 0.366<br>32 | -0.182<br>32 | 0.153<br>32 | 0.049<br>32 | 0.139<br>32 | -0.387<br>32 | -0.283<br>32 |
| Trait 9: GSE16780_UCLA_ML0911::1417836_at_A<br>Gpx7 on Chr 4 @ 108.073058 Mb<br>glutathione peroxidase 7 | -0.223<br>32 | -0.037<br>32 | 0.046<br>32 | 0.270<br>32 | -0.099<br>32 | -0.058<br>32 | 0.021<br>32 | -0.076<br>32 | ***n***<br>32 | 0.349<br>32 | -0.422<br>32 | -0.023<br>32 | 0.199<br>32 | 0.161<br>32 | 0.185<br>32 | 0.238<br>32 | 0.280<br>32 |
| Trait 10: GSE16780_UCLA_ML0911::1424099_at_A<br>Gpx8 on Chr 13 @ 113.833243 Mb<br>glutathione peroxidase 8<br>last 2 exons and proximal 3' UTR | -0.296<br>32 | -0.186<br>32 | 0.239<br>32 | 0.014<br>32 | 0.472<br>32 | 0.429<br>32 | -0.078<br>32 | -0.012<br>32 | 0.462<br>32 | ***n***<br>32 | 0.125<br>32 | -0.235<br>32 | 0.124<br>32 | 0.220<br>32 | 0.226<br>32 | 0.241<br>32 | 0.007<br>32 |
| Trait 11: GSE16780_UCLA_ML0911::1451124_at_A<br>Sod1 on Chr 16 @ 90.223027 Mb<br>superoxide dismutase 1, soluble<br>first four exons and 3' UTR | 0.433<br>32 | 0.376<br>32 | 0.503<br>32 | -0.555<br>32 | 0.428<br>32 | 0.490<br>32 | 0.008<br>32 | 0.269<br>32 | -0.362<br>32 | 0.107<br>32 | ***n***<br>32 | -0.363<br>32 | 0.397<br>32 | 0.401<br>32 | 0.124<br>32 | -0.261<br>32 | -0.500<br>32 |
| Trait 12: GSE16780_UCLA_ML0911::1435304_at_A<br>Sod1 on Chr 16 @ 90.22488 Mb<br>superoxide dismutase 1, soluble<br>antisense in intron 3 and exon 4 | -0.028<br>32 | -0.052<br>32 | -0.087<br>32 | 0.092<br>32 | -0.187<br>32 | -0.151<br>32 | -0.213<br>32 | -0.116<br>32 | -0.097<br>32 | -0.224<br>32 | -0.212<br>32 | ***n***<br>32 | -0.113<br>32 | -0.203<br>32 | -0.053<br>32 | -0.034<br>32 | 0.103<br>32 |

| | | | | | | | | | | | | | | | | | |
|---|---|---|---|---|---|---|---|---|---|---|---|---|---|---|---|---|---|
| Trait 13: GSE16780_UCLA_ML0911::1417193_at_A<br>Sod2 on Chr 17 @ 13.206311 Mb<br>superoxide dismutase 2, mitochondrial<br>last three exons and proximal 3' UTR | 0.500<br>32 | 0.678<br>32 | 0.467<br>32 | -0.426<br>32 | 0.328<br>32 | 0.449<br>32 | 0.307<br>32 | 0.325<br>32 | 0.024<br>32 | 0.076<br>32 | 0.549<br>32 | -0.124<br>32 | ***n***<br>32 | 0.906<br>32 | 0.114<br>32 | -0.134<br>32 | -0.355<br>32 |
| Trait 14: GSE16780_UCLA_ML0911::1448610_a_at_A<br>Sod2 on Chr 17 @ 13.208027 Mb<br>superoxide dismutase 2, mitochondrial<br>last exon and proximal 3' UTR | 0.356<br>32 | 0.522<br>32 | 0.525<br>32 | -0.310<br>32 | 0.422<br>32 | 0.586<br>32 | 0.433<br>32 | 0.214<br>32 | 0.039<br>32 | 0.205<br>32 | 0.514<br>32 | -0.206<br>32 | 0.909<br>32 | ***n***<br>32 | 0.025<br>32 | -0.020<br>32 | -0.249<br>32 |
| Trait 15: GSE16780_UCLA_ML0911::1417194_at_A<br>Sod2 on Chr 17 @ 13.20865 Mb<br>superoxide dismutase 2, mitochondrial | 0.219<br>32 | 0.200<br>32 | -0.094<br>32 | -0.295<br>32 | 0.049<br>32 | -0.153<br>32 | -0.237<br>32 | 0.314<br>32 | 0.204<br>32 | 0.168<br>32 | 0.246<br>32 | -0.066<br>32 | 0.244<br>32 | 0.123<br>32 | ***n***<br>32 | 0.140<br>32 | -0.216<br>32 |
| Trait 16: GSE16780_UCLA_ML0911::1417634_at_A<br>Sod3 on Chr 5 @ 52.759404 Mb<br>superoxide dismutase 3, extracellular | -0.401<br>32 | -0.011<br>32 | 0.022<br>32 | -0.081<br>32 | 0.082<br>32 | -0.105<br>32 | 0.193<br>32 | -0.281<br>32 | 0.141<br>32 | 0.207<br>32 | -0.225<br>32 | -0.244<br>32 | -0.115<br>32 | -0.045<br>32 | -0.057<br>32 | ***n***<br>32 | 0.346<br>32 |
| Trait 17: GSE16780_UCLA_ML0911::1417633_at_A<br>Sod3 on Chr 5 @ 52.760191 Mb<br>superoxide dismutase 3, extracellular | -0.362<br>32 | -0.326<br>32 | -0.400<br>32 | 0.295<br>32 | -0.407<br>32 | -0.276<br>32 | 0.163<br>32 | -0.260<br>32 | 0.082<br>32 | -0.055<br>32 | -0.465<br>32 | 0.084<br>32 | -0.542<br>32 | -0.409<br>32 | -0.402<br>32 | 0.400<br>32 | ***n***<br>32 |

Supplementary Table s2. Spearman rank correlation of three oxidative genes among humans (Human liver)

Spearman Rank Correlation (rho)

| | | Trait1 | Trait2 | Trait3 | Trait4 | Trait5 | Trait6 | Trait7 | Trait8 | Trait9 | Trait10 | Trait11 | Trait12 | Trait13 | Trait14 | Trait15 |
|---|---|---|---|---|---|---|---|---|---|---|---|---|---|---|---|---|
| Pearson r | Trait 1: HLC_0311::10023826334<br>CAT on Chr 11 @ 34.493607 Mb<br>catalase | ***n***<br>427 | -0.488<br>427 | -0.473<br>427 | -0.458<br>427 | 0.516<br>427 | 0.077<br>427 | -0.149<br>426 | 0.152<br>426 | 0.008<br>427 | -0.336<br>427 | -0.415<br>427 | 0.800<br>426 | 0.849<br>427 | -0.712<br>427 | -0.237<br>427 |
| | Trait 2: HLC_0311::10023814607<br>GPX1 on Chr 3 @ 49.394608 Mb<br>glutathione peroxidase 1 | -0.438<br>427 | ***n***<br>427 | 0.467<br>427 | 0.495<br>427 | -0.093<br>427 | -0.361<br>427 | 0.195<br>426 | -0.063<br>426 | -0.146<br>427 | 0.302<br>427 | 0.340<br>427 | -0.535<br>426 | -0.519<br>427 | 0.391<br>427 | -0.058<br>427 |
| | Trait 3: HLC_0311::10023809881<br>GPX2 on Chr 14 @ 65.405871 Mb<br>glutathione peroxidase 2 (gastrointestinal) | -0.359<br>427 | 0.463<br>427 | ***n***<br>427 | 0.332<br>427 | -0.073<br>427 | -0.306<br>427 | 0.207<br>426 | -0.174<br>426 | -0.134<br>427 | 0.263<br>427 | 0.417<br>427 | -0.330<br>426 | -0.366<br>427 | 0.337<br>427 | 0.032<br>427 |
| | Trait 4: HLC_0311::10023805643<br>GPX3 on Chr 5 @ 150.408554 Mb | -0.388<br>427 | 0.500<br>427 | 0.616<br>427 | ***n***<br>427 | -0.019<br>427 | -0.233<br>427 | 0.285<br>426 | -0.037<br>426 | -0.181<br>427 | 0.127<br>427 | 0.050<br>427 | -0.430<br>426 | -0.443<br>427 | 0.440<br>427 | 0.180<br>427 |

| | | | | | | | | | | | | | | | |
|---|---|---|---|---|---|---|---|---|---|---|---|---|---|---|---|
| glutathione peroxidase 3 (plasma) | | | | | | | | | | | | | | | |
| Trait 5: HLC_0311::10023815372<br>GPX4 on Chr 19 @ 1.106787 Mb<br>glutathione peroxidase 4 (phospholipid hydroperoxidase) | 0.459<br>427 | -0.022<br>427 | 0.139<br>427 | 0.215<br>427 | ***n***<br>427 | -0.273<br>427 | -0.157<br>426 | -0.091<br>426 | -0.376<br>427 | -0.303<br>427 | -0.379<br>427 | 0.385<br>426 | 0.437<br>427 | -0.451<br>427 | -0.200<br>427 |
| Trait 6: HLC_0311::10023813546<br>GPX5 on Chr 6 @ 28.502728 Mb<br>glutathione peroxidase 5 (epididymal androgen-related protein) | 0.066<br>427 | -0.398<br>427 | -0.401<br>427 | -0.366<br>427 | -0.264<br>427 | ***n***<br>427 | -0.076<br>426 | 0.149<br>426 | 0.612<br>427 | 0.098<br>427 | 0.129<br>427 | 0.168<br>426 | 0.091<br>427 | -0.096<br>427 | 0.129<br>427 |
| Trait 7: HLC_0311::10023819620<br>GPX5 on Chr 6 @ 28.502728 Mb<br>glutathione peroxidase 5 (epididymal androgen-related protein) | -0.088<br>426 | 0.097<br>426 | 0.207<br>426 | 0.179<br>426 | -0.017<br>426 | -0.004<br>426 | ***n***<br>426 | 0.131<br>425 | 0.022<br>426 | 0.102<br>426 | 0.059<br>426 | -0.030<br>425 | -0.061<br>426 | 0.230<br>426 | -0.162<br>426 |
| Trait 8: HLC_0311::10025911877<br>GPX6 on Chr 6 @ 28.471072 Mb<br>glutathione peroxidase 6 (olfactory) | 0.139<br>426 | -0.090<br>426 | -0.236<br>426 | -0.142<br>426 | -0.105<br>426 | 0.191<br>426 | -0.102<br>425 | ***n***<br>426 | 0.225<br>426 | 0.066<br>426 | 0.019<br>426 | 0.174<br>425 | 0.174<br>426 | -0.026<br>426 | -0.077<br>426 |
| Trait 9: HLC_0311::10023821927<br>GPX7 on Chr 1 @ 53.074723 Mb<br>glutathione peroxidase 7 | 0.143<br>427 | -0.312<br>427 | -0.518<br>427 | -0.660<br>427 | -0.439<br>427 | 0.603<br>427 | -0.109<br>426 | 0.214<br>426 | ***n***<br>427 | 0.214<br>427 | 0.337<br>427 | 0.069<br>426 | -0.007<br>427 | -0.075<br>427 | 0.148<br>427 |

| | | | | | | | | | | | | | | | |
|---|---|---|---|---|---|---|---|---|---|---|---|---|---|---|---|
| Trait 10: HLC_0311::10025907159<br>GSR on Chr 8 @ 30.535579 Mb<br>glutathione reductase | -0.296<br>427 | 0.268<br>427 | 0.077<br>427 | -0.062<br>427 | -0.272<br>427 | 0.100<br>427 | 0.037<br>426 | 0.004<br>426 | 0.252<br>427 | ***n***<br>427 | 0.513<br>427 | -0.204<br>426 | -0.243<br>427 | 0.284<br>427 | -0.077<br>427 |
| Trait 11: HLC_0311::10025903928<br>GSS on Chr 20 @ 33.516235 Mb<br>glutathione synthetase | -0.353<br>427 | 0.297<br>427 | 0.240<br>427 | -0.071<br>427 | -0.346<br>427 | 0.140<br>427 | 0.032<br>426 | 0.015<br>426 | 0.329<br>427 | 0.487<br>427 | ***n***<br>427 | -0.293<br>426 | -0.354<br>427 | 0.177<br>427 | 0.126<br>427 |
| Trait 12: HLC_0311::10023815327<br>SOD1 on Chr 21 @ 33.041243 Mb<br>superoxide dismutase 1, soluble (amyotrophic lateral sclerosis 1 (adult)) | 0.803<br>426 | -0.512<br>426 | -0.348<br>426 | -0.514<br>426 | 0.283<br>426 | 0.220<br>426 | -0.048<br>425 | 0.150<br>425 | 0.344<br>426 | -0.124<br>426 | -0.198<br>426 | ***n***<br>426 | 0.962<br>426 | -0.575<br>426 | -0.224<br>426 |
| Trait 13: HLC_0311::10033668826<br>SOD1 on Chr 21 @ 33.041243 Mb<br>superoxide dismutase 1, soluble (amyotrophic lateral sclerosis 1 (adult)) | 0.842<br>427 | -0.492<br>427 | -0.369<br>427 | -0.524<br>427 | 0.324<br>427 | 0.152<br>427 | -0.065<br>426 | 0.155<br>426 | 0.292<br>427 | -0.161<br>427 | -0.252<br>427 | 0.971<br>426 | ***n***<br>427 | -0.585<br>427 | -0.254<br>427 |
| Trait 14: HLC_0311::10023816501<br>SOD2 on Chr 6 @ 160.102754 Mb<br>superoxide dismutase 2, mitochondrial | -0.696<br>427 | 0.372<br>427 | 0.249<br>427 | 0.327<br>427 | -0.416<br>427 | -0.092<br>427 | 0.099<br>426 | -0.041<br>426 | -0.119<br>427 | 0.264<br>427 | 0.120<br>427 | -0.548<br>426 | -0.554<br>427 | ***n***<br>427 | 0.078<br>427 |

| | | | | | | | | | | | | | | | |
|---|---|---|---|---|---|---|---|---|---|---|---|---|---|---|---|
| Trait 15: HLC_0311::10023810228<br><br>SOD3 on Chr 4 @ 24.802467 Mb<br><br>superoxide dismutase 3, extracellular | -0.219<br>427 | -0.091<br>427 | 0.018<br>427 | 0.133<br>427 | -0.180<br>427 | 0.086<br>427 | -0.038<br>426 | -0.095<br>426 | 0.076<br>427 | -0.080<br>427 | 0.113<br>427 | -0.235<br>426 | -0.254<br>427 | 0.021<br>427 | ***n***<br>427 |
